# Simulated Microgravity Condition Alters the Gene Expression of some ECM and Adhesion Molecules in Adipose Derived Stem Cells

**DOI:** 10.22088/IJMCM.BUMS.7.3.146

**Published:** 2018-10-08

**Authors:** Farid Ebnerasuly, Zahra Hajebrahimi, Seyed Mehdi Tabaie, Mojtaba Darbouy

**Affiliations:** 1 *Department of Biology, Fars Science and Research Branch, Islamic Azad University, Marvdasht, Iran.*; 2 *Department of Biology, Marvdasht Branch, Islamic Azad University, Marvdasht, Iran.*; 3 *Aerospace Research Institute, Ministry of Science Research and Technology, Tehran, Iran.*; 4 *Department of Photo Healing and Regeneration, Medical Laser Research Center, Yara Institute, ACECR, Tehran, Iran*

**Keywords:** Adipose- derived stem cells, simulated microgravity, extracellular matrix, adhesion molecules

## Abstract

Adipose- derived stem cells (ADSCs) are widely used for tissue engineering and regenerative medicine. The beneficial effects of ADSCs on wound healing have already been reported. Remodeling of extracellular matrix (ECM) is the most important physiological event during wound healing. ECM is sensitive to mechanical stresses and the expression of its components can be therefore influenced. The aim of this study was to investigate the effect of simulated microgravity on gene expression of some ECM and adhesion molecules in human ADSCs. After isolation and characterization of ADSCs, cells were exposed to simulated microgravity for 1, 3 and 7 days. Real-time PCR, fluorescence immunocytochemistry, and MTT assay were performed to evaluate the alterations of integrin subunit beta 1 (*ITGB1*), collagen type 3 (*ColIII*), matrix metalloproteinase-1 (*MMP1*), *CD44*, fibrillin (*FBN1*), vimentin (*VIM*) genes, and ColIII protein levels as well as cells viability. Microgravity simulation increased the expression of *ITGB1, ColIII, MMP1*, and *CD44 *and declined the expression of *FBN1 *and* VIM* genes. ColIII protein levels also increased. There were no significant changes in the viability of cells cultured in microgravity. Since the high expression of ECM components is known as one of the fibroblast markers, our data suggest that pretreatment of ADSCs by simulated microgravity may increase their differentiation capacity towards fibroblastic cells. Microgravity had not adversely affected the viability of ADSCs, and it is likely to be used alone or in combination with biochemical inducers for cell manipulation.

Mesenchymal stem cells (MSCs) are multipotent stromal cells that can be differentiated into a variety of mesenchymal tissues, including cartilage, bone, fat, and several other tissues (1). Bone marrow stromal cells (BMSCs) are the most considered source of MSCs for therapeutic purposes since they have been introduced in the 1960s (2). After BMSCs finding, MSCs have been demonstrated to exist in almost all tissues in the body such as adipose tissue, umbilical cord blood, peripheral blood, dermis, amniotic fluid, and even in tumors (3-7). For the first time, adipose-derived stem cells (ADSCs) were introduced in 2001 as MSCs (8). ADSCs are propounded as a great source of MSCs that are easily achievable from adipose tissue via liposuction. In recent years, ADSCs are widely used for tissue engineering and regenerative medicine instead of using BMSCs because of their abundance in adipose tissue with minimal mortality, easy availability, and safe isolation (9). It also has been shown that ADSCs have therapeutic effects in wound healing and tissue repair studies (10). Today, impaired wound healing is challenging because of inadequate skin tissue in the site of injury. Therefore, stem cell biology provides the novel option for the cell therapy of wound repair.

Remodeling of the extracellular matrix (ECM) is the most important physiological event during the wound healing process (11). ECM is the largest component of the normal skin, and its components play several key functions in wound healing process, such as providing support to lead cells into the injury area, and stimulating cells to proliferate and differentiate (11). Apart from the role of ECM in wound healing, it is also involved in a series of other cell activities including signal- transduction pathways, cell migration, and organization of cells into tissues and coordination of cell functions (12, 13).

Studies have shown that the expression of ECM components can be influenced by mechanical stress (14). One of the most important mechanical factors that affect all types of life on earth is gravity. Previous reports have indicated that cultured cells like MSCs have responded to gravity (both microgravity and hypergravity), too (15, 16).

Microgravity has been confirmed to affect growth and physiology of cell through impacting on intracellular signaling mechanisms, cell secretions, and gene expression (17, 18). It has been shown that components of the cytoskeleton such as actin polymer are gravity sensitive and reorganized in microgravity condition. This can lead to changes in cell morphology and fate (19, 20). Integrin is a mediated cell adhesion protein that connects the cytoskeleton to ECM (21). Vimentin, integrin, and CD44 are the proteins that play important roles in cell adhesion and ECM formation (22). With regard to ECM roles and the communication between ECM and cytoskeleton, it is expected that changes in mechanical forces have a significant effect on the ECM structure. Most researchers have used BMSCs to study the effect of simulated microgravity condition on the function and structure of stem cells (23, 24). According to the variety in cell types and differences in their structure and functions, the effects of gravity on various cell types are different. Therefore, molecular mechanisms of the cell response to gravity are not fully understood yet. Altogether, the aim of the present study was to investigate the effect of simulated microgravity condition on gene expression of some components of ECM and adhesion molecules. Our selected genes were integrin subunit beta 1 (*ITGB1*), collagen type 3 (*ColIII*), fibrillin (*FBN1*), vimentin (*VIM*), matrix metalloproteinase-1 (*MMP1*), and *CD44*. We have examined the expression of *ColIII* at RNA and protein levels Under microgravity simulated by clinostat.

## Materials and methods

**Preparation of adipose tissue sample**

All experiments were performed according to the Clinical Research Ethics Committee of the Medical Laser Research Center, ACECR, Tehran, Iran (Ethical Code: IR. ACECR. ROYAN. REC. 1395.54). Tissue samples were acquired from 2 donors (34 and 48 years old). Each human subject signed a consent form. Adipose samples were obtained from patients going through cosmetic liposuction. After receiving, blood phase was removed, and the rest of the samples were washed with Hank's Balanced Salt Solution (HBSS;Biowest, France) and used for the cell isolation.

**Isolation of ADSCs**

Cell isolation was done by enzymatic digestion method as described by Zuk et al. (4). Briefly, the adipose samples were incubated at 37 ºC for 60 min in HBSS containing 1 mg/ml of collagenase type I (Sigma, USA), and shacked every 15 min. After diluting with an equal volume of serum (Biowest, France) -containing medium (Dulbecco's Modified Eagle Medium) (DMEM; Biowest, France), the suspension was centrifuged at 400 g for 10 min, and floated lipid layer was discarded. The stromal vascular fraction (SVF) was washed and resuspended in DMEM medium supplemented with 10% fetal bovine serum (FBS), antibiotic-antimycotic (Biowest, France) solution and seeded into a 25 cm^2^ cell-culture flask (TPP, Switzerland) and kept at 37 °C and 5% CO2 incubator. After 48 h, the non-adherent cells and cellular debris were discarded by changing the medium and the adherent cells were preserved to achieve ~80% confluence. The cells were passaged by a standard trypsinization (Biowest, France) protocol and cells at passages 3– 4 were used for the analysis of ADSC surface markers and differentiation experiments.

**Characterization of ADSCs**

The isolated cells from passage 3 were suspended in phosphate-buffered saline (PBS) (3×10^5^/100 µl for each reaction) and then incubated for 30 min at 4 ºC in the dark with the PE (phycoerythrin) and FITC-conjugated (fluorescein isothiocyanate) antibodies (BD Biosciences PharMingen, USA) against CD90-PE, CD105-FITC, CD73-PE as positive markers, and CD34-PE and CD45-FITC as negative markers according to Dominici et al. (1). Flow cytometric analyses were carried out using a Cyflow Space (Partec, Germany) flow cytometer. Data were then analyzed by the FloMax software (version 2.70).

**Functional characterization of ADSCs**

ADSCs are multipotent stem cells that have the capacity to differentiate into osteoblasts, adipocytes, and chondrocytes (1). In this present work, to examine the multipotent potential of the isolated cells, adipogenic and osteogenic differentiation was performed using Human Mesenchymal Stem Cell Functional Identification Kit (R&D systems, USA) according to the manufacturer’s recommendations. Briefly, for adipocyte differentiation, ADSCs were seeded into a 6-well culture plate (2.1×10^4^ cells per cm^2^) containing α-MEM media (Biowest, France) supplemented with antibiotics and 10% FBS, and allowed to achieve 90-100% confluence. Then the medium was removed and the adipogenic differentiation medium (α-MEM, hydrocortisone, 3-isobutyl-1-methylxanthine, and indomethacin) was added to pellets. Differentiation medium was replaced every 3 days. After 9 days, lipid vacuoles were observed and confirmed by Oil Red O (Sigma, USA) staining.

Oil Red O staining was done as described by Aldridge et al. (25). Briefly, cells were washed with PBS and fixed in neutral buffered formalin (10%) for 30 min at room temperature. Then the cells were washed with distilled water and incubated with enough 60% isopropanol at room temperature for 5 min. After isopropanol removal, oil red solution [0.5% oil red (w/v) in isopropanol] was added for 15 min and incubated at room temperature. Then oil red was removed, cells were washed with distilled water, and were observed under a light microscope.

For osteoblast differentiation, ADSCs were seeded in a 6-well culture plate containing α-MEM supplemented with antibiotics and 10% FBS to achieve 50-70% confluence. Then the medium was removed and the osteogenic differentiation medium (α-MEM, dexamethasone, ascorbic acid-2-phosphate, and β-glycerophosphate) was added to pellets. Differentiation medium was changed every 3 days. After 15 days, cells started to detach and osteocytes were prepared. Accumulation of calcium was confirmed by Alizarin Red S staining.

Alizarin Red S staining was done as described by Fan et al. (26). Briefly, cells were washed with PBS and fixed in neutral buffered formalin (10%) for 15 min at room temperature. Then cells were washed with distilled water and incubated in 2% Alizarin Red staining solution at room temperature for 5 min with gentle shaking. Then dye was removed, cells were washed with distilled water, and were observed under a light microscope. The red staining indicates accumulation of calcium in differentiated cells.

**Microgravity simulation**

Clinostat (donation from the United Nations Office at Vienna; office for outer space affairs) was used for simulating microgravity. This device prevents cell sense gravity by rotating, so the gravity vector is not recognizable to cells. For this purpose, clinostat was sterilized by UV and ethanol (70%) and put in a 37 °C CO2 incubator. Then, ADSCs were cultured in a 12.5 cm^2 ^cell-culture flask. After cell adhesion, flasks were filled completely by medium to prevent the presence of air bubbles. To maintain the pH balance, the medium was supplemented with 15 mM 4-(2-hydroxyethyl) -1- piperazineethanesulfonic acid (HEPES). Samples were fixed at the center of the clinostat. The clinostat rotation speed was 30 rpm (27). The rotation times were 1, 3 and 7 days.

**MTT assay**

MTT assay was done as described previously (28). A working solution of 5 mg/mL (3-(4, 5-dimethylthiazol-2-yl) -2, 5-diphenyl tetrazolium bromide) (MTT; Atocel, Austria) dissolved in PBS was added to the culture medium (100 µl/well) to detect cell viability. After 3 h incubation at 37 °C, purple crystals of formazan were observed. The medium was removed and to solve formazan, 100 µl dimethyl sulfoxide (DMSO) (Atocel, Austria) was added to each well. The amount of formazan was quantitated with an ELISA plate reader (BioTech, USA), at 570 nm wavelength.

**Real-time quantitative PCR**

The expression of selected genes was analyzed in all samples by real-time RT-PCR as described previously (29). Briefly, total RNA was isolated from samples using RNA isolation kit (Cell Amp^TM^ Direct RNA Prep Kit for RT-PCR; Takara, Japan). Prime Script^TM^ RT reagent Kit (Takara, Japan) was used for cDNA synthesis according to the manufacturer’s recommendations. Quantitative real-time RT-PCR was done using StepOnePlus Real-Time PCR (Applied Biosystems, USA). PCR program was: initial denaturation at 95º C for 2 min; followed by 40 cycles of denaturation at 95 ºC for 5 s and annealing at 60 ºC for 30 s. Agarose gel electrophoresis and melt curves for all genes were obtained to check PCR reaction for the presence of nonspecific products and confirm the specificity of the reaction. Changes in the fold number were evaluated using the 2^-ΔΔCt^ method. The expression of glyceraldehyde-3-phosphate dehydrogenase gene (*GAPDH*) of each sample was measured as a normalization control. Specific primers were designed using Oligo7 primer analysis software and then submitted to BLAST search to ensure that the sequences were specific just for the gene of interest, and were synthesized by Macrogen (South Korea) as mentioned in Table 1.

**Table 1:** Primers for quantitative real-time PCR

Gene	Forward and Reverse primer (5′–3′)	Gene Bank No.	Amplicon size(bp)
*GAPDH*	F: AACAGCCTCAAGATCATCAGCAA R: GATGGCATGGACTGTGGTCAT	NM_001289746.1	120
*ITGB1*	F: TGCACCAGCCCATTTAGCTAC R: CCTCCAGCCAATCAGTGATCC	NM_033668.2	168
*CD44*	F: GCTTCAATGCTTCAGCTCCAC R: GGGTTGCTGGGGTAGATGTC	NM_000610.3	166
*MMP1*	F: TCACACCTCTGACATTCACCAAG R: TCCCGATGATCTCCCCTGAC	NM_002421.3	79
*VIM*	F: TGAAAGTGTGGCTGCCAAGA R: CAGAGAGGTCAGCAAACTTGGA	NM_001278918.1	71
*COL3A1*	F: GGATGGTTGCACGAAACACAC R: CAGGACCACCAATGTCATAGGG	NM_000090.3	116
*FBN1*	F: GAGTGCCTTGACAATCGGGA R: GATTTGGTGACGGGGTTCCT	NM_000138.4	95

**Fig. 1:**
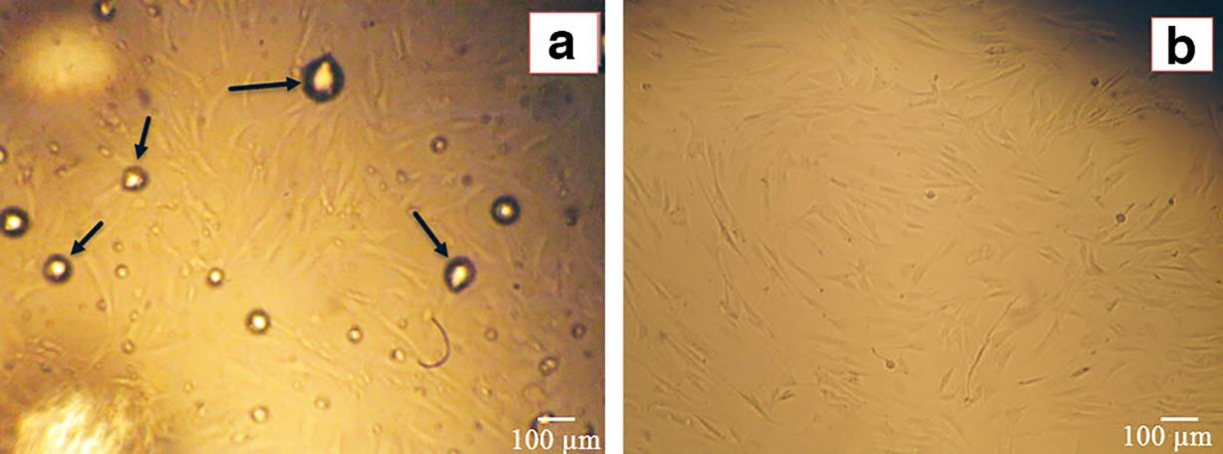
Isolated ADSCs with the enzymatic method from lipoaspirate samples. (A). Following a 48- hour SVF culture, fibroblast-like spindle shape cells can be clearly observed (100x).The arrows indicate the remains of oil spots from digested fat. (B). After changing medium, ADSCs at 40-50% confluence were observed. Magnification 100 x

**Fig. 2:**
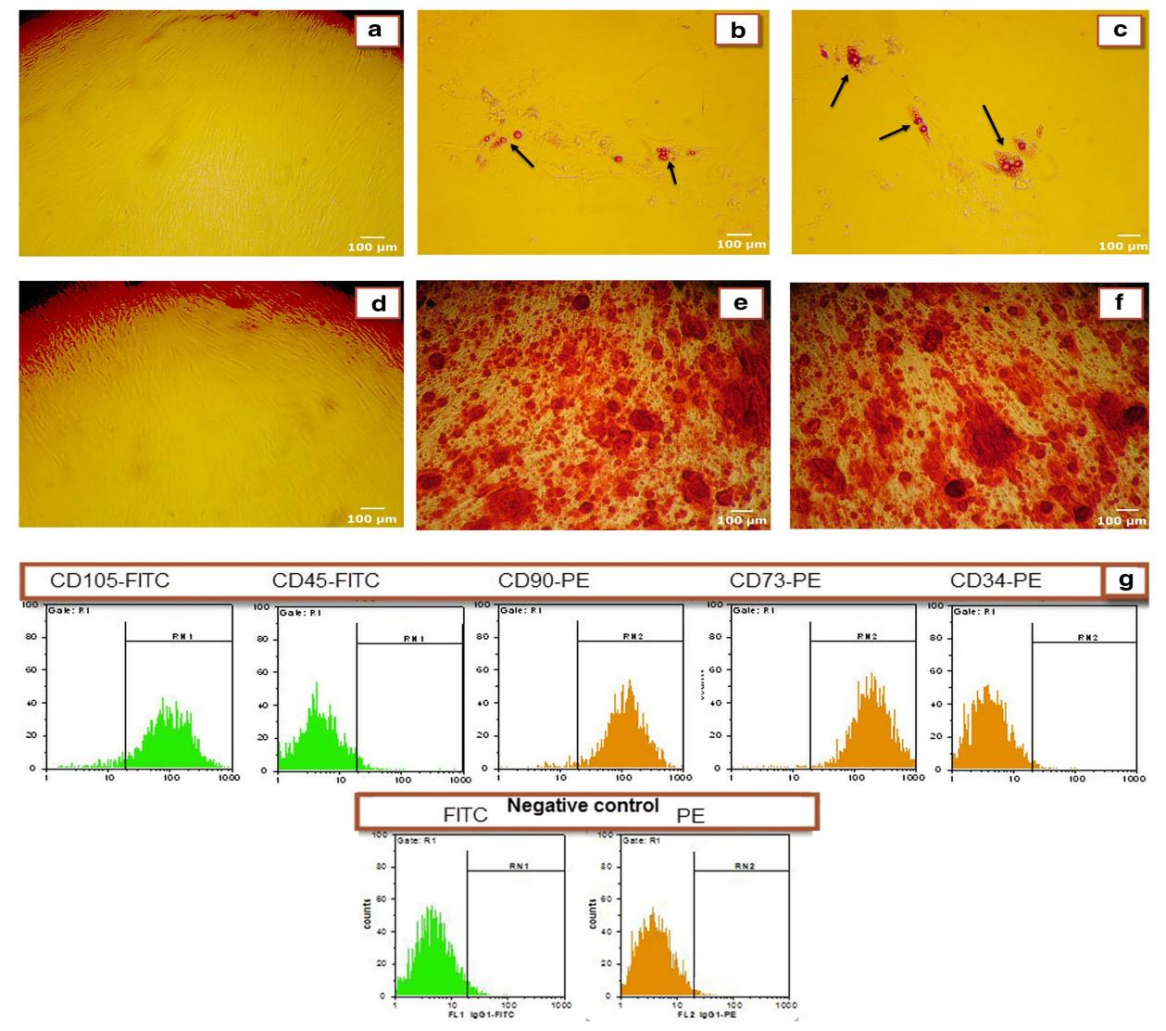
Characterization of ADSCs. Figures A to F show the ability of human ADSCs to differentiate into adipocyte and osteoblast lineages (100x). (A and D) ADSCs cultured in control media; (B and C) ADSCs cultured in the presence of adipogenic inducer for 9 days and stained with Oil Red O dye. Arrows show the accumulation of lipid vacuoles indicating differentiation to adipogenic cell lineage; (E and F) ADSCs cultured in the presence of osteogenic inducer for 15 days and stained with Alizarin Red S dye. Production of orange-red calcium deposits demonstrated the successful differentiation of ADSCs to osteogenic cell lineage. (G) Immunophenotypic characterization of human MSCs were carried out using flow cytometry. Third-passage of isolated ADSCs was positive for MSCs markers including CD90, CD73 and CD105 and negative for hematopoietic markers including CD34 and CD45 (for all the donors)

**Fig. 3:**
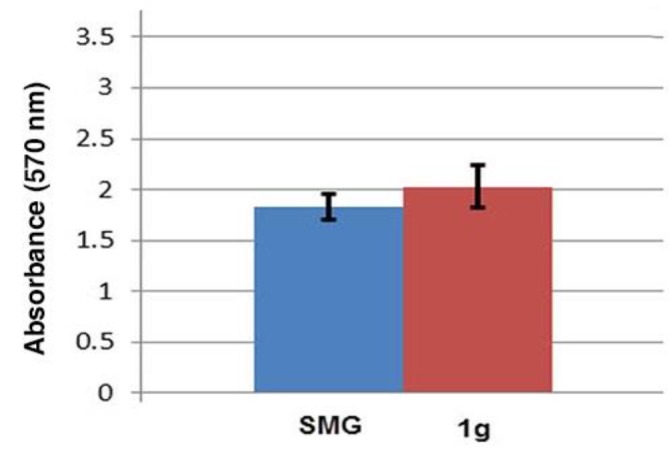
Cell viability of ADSCs cultured in simulated microgravity (SMG) and static culture after 7 days. There were no statistically significant changes in viability between two culture conditions (n=3)

**Fig. 4:**
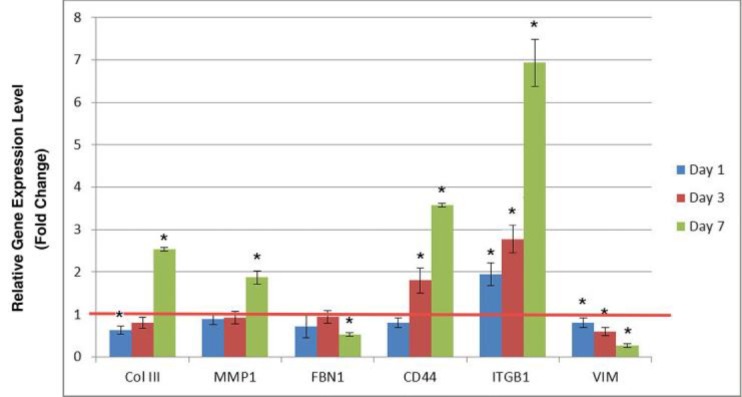
Relative gene expression analysis of some ECM and adhesion molecules under simulated microgravity condition for the 1, 2 and 7 days in comparison to the control group in normal gravity (n=3). Gene's expressions were normalized to GAPDH in the same samples. Values are mean ± staandard deviation; *indicates P<0.05

**Fig. 5:**
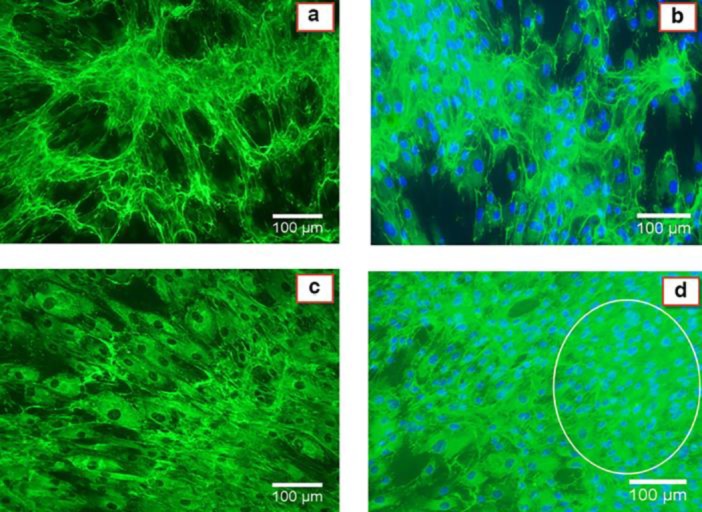
Fluorescence immunocytochemistry analysis of ColIII. (A and B) Anti-ColIII staining in cells cultured in normal or 1g condition. (C and D) Anti- ColIII staining in cells cultured in simulated microgravity condition for 7 days. (A and C) Anti- ColIII staining without Hoechst treatment. (B and D) Anti-ColIII staining plus Hoechst treatment. The circle shows cell aggregated production. ImageJ was used to quantify the COLIII-positive area in six randomly chosen fields for each experiment (n =3). P<0.05 was considered statistically significant. Magnification 100x.

**Fluorescence immunocytochemistry**

To analyze the protein expression of collagen type 3 (COLIII), fluorescence immunocyto-chemistry was carried out on non-treated ADSCs (cells cultured in normal gravity) and on ADSCs cultured in simulated microgravity condition for 7 days. For this purpose, cells were fixed with 4% paraformaldehyde for 20 min at room temperature. Following a wash with ice-cold PBS (three times), cells were permeabilized by exposure to 0.01% Triton X-100 (Sigma, USA) at room temperature for 20 min and washed with PBS three times. Blocking was done with normal goat serum (10%) and BSA (1%) in PBS for 20 min and at room temperature. Then cells were incubated with the anti-rabbit COL3A1 primary antibody (1:100; Biorbyt, UK) overnight at 4 °C. Following a wash, anti-rabbit IgG Alexa Fluor fluorescent secondary antibody (5 µg/ml; Invitrogen, USA) was added for 1 h at room temperature in the dark. Subsequently, the cells were treated with Hoechst 33342 (dilution 1:100; Sigma, USA) for 10 min. Images were obtained using a fluorescent microscope (Olympus, Japan). Quantification of COLIII-positive area was performed using ImageJ 1.49v software (National Institute of Health, USA; http://imagej.nih.gov/ij).

**Statistical analysis**

To avoid errors due to differences in sample source, for each assay and analysis, cells from the same donor were used. For each assay, three cultures were prepared, using cells from the same donor. The numbers of experiments carried out are indicated in the respective figure legends. Relative expression was evaluated using REST 2009 software (Version 2.0.13). Statistical correlation was performed using independent samples t-test and P<0.05 was considered statistically significant. All experiments were replicated at least twice.

## Results

**Isolation and culture of ADSCs**

 After 48 h of culture of SVF in cell culture flask, Fibroblast-like spindle shape cells were observed (Figure 1). The adherent cells proliferated at a considerable rate and reached 70-80% confluence after 4 days. Both the shape and adhesion properties of the cells confirmed that the isolated cells were MSCs.

**Flow cytometry analysis**

As shown in figure 2, flow cytometric analysis of ADSCs showed that a higher percentage of ADSCs (>95%) expressed CD90, CD73, and CD105 (surface antigens of MSCs) but did not expressed CD34 and CD45 (hematopoietic markers) (Figure 2G). These results showed that the isolated cells were MSCs.

**Differentiation of isolated cells to adipocyte and osteoblast**

To demonstrate the pluripotent capacity of ADSCs, they were differentiated into adipocyte and osteocyte. Staining results by Oil Red O (Figure 2B and 2C) and Alizarin Red S (Figure 2E and 2F) confirmed that the isolated cells were differentiated well into adipocyte and osteoblast lineages (Figure 2).

**Cell viability assay**

The effect of simulated microgravity on ADSCs viability was determined using MTT assay. As shown in Figure 3, 7 days of simulated microgravity had no significant effect on the viability of the ADSCs (P>0.05).

**Gene expression analysis by real-time PCR**

We employed a real-time PCR method to measure the effect of simulated microgravity on gene expression of ECM and adhesion molecules including *ColIII*, *MMP1*, *FBN1*, *CD44*, *ITGB1*, and *VIM*. As shown in Figure 4, simulated microgravity increased the expression of *ColIII*, *MMP1*, *CD44*, and *ITGB1*. Our result showed a modest decline in the expression of *ColIII *(about 40%) one day after simulated microgravity treatment. The level of *ColIII *expression was gradually up-regulated by 2 days after simulated microgravity treatment, and increased up to 2.5 fold of the control level after 7 days of exposure to simulated microgravity condition. We could not observe any changes in *MMP1* expression following microgravity simulation for 1 and 2 days. Similar to *ColIII*, 7 days culture in simulated microgravity increased the expression of *MMP1* up to 2 fold of the control level. A similar result was observed for *CD44* expression. 1 day simulated microgravity induction had no effect on* CD44* expression. However, its expression increased up to 2 fold of the control level at 2 days after microgravity simulation, and enhanced up to 3.5 fold of the control group by 7 days. In contrast to *ColIII*, the expression of *ITGB1* increased up to 2 fold of the control level as early as 1 day after microgravity simulation, followed by a marked up-regulation by 7 days, when the expression showed a 7 fold elevation in comparison to the control sample.

Unlike the genes described above, simulated microgravity decreased the expression of *FBN1* and *VIM*. The expression of *VIM* showed a significant reduction (about 85%) 7 days after simulated microgravity induction. A similar reduction of expression was obtained for *FBN1*. The expression of *FBN1* decreased 50% following 7 days exposure of ADSCs to simulated microgravity.

**Fluorescence immunocytochemistry**

We analyzed the expression of collagen type III at the protein level using immunocytochemistry assay. Quantification of COLIII-positive area was performed using ImageJ 1.49v software. As seen in Figure 5, the expression of COLIII in simulated microgravity environment (Figure 5C and 5D) is significantly higher than its expression in 1g condition (Figure 5A and 5B) (56.96±2.30, 81.01±7.96, 40.88±4.07, and 60.12±4.40 for Fig. 5C, 5D, 5A and 5B, respectively; P<0.05). Also, it seems that simulated microgravity condition induced cells to generate aggregated and accumulated structure.

## Discussion

Gravity is one of the most important mechanical factors on earth that has extensive effects on the evolution and physiology of all living organisms. Under simulated microgravity condition, the cell morphology switches from a flattened spindle shape to an almost round phenotype. This is the result of the destruction of microfilament structures of the cytoskeleton including actin polymers. Such changes may affect intracellular signal cascades and can lead to changes in cell differentiation capability (19, 20). Thus, simulated microgravity may be used as a novel methodology for manipulating cells along with other biochemical techniques (30, 31).

In this study, we found that simulated microgravity condition alters the gene expression of some important ECM and adhesive molecules in ADSCs. Clinostat was used to simulate the microgravity environment. Our results showed that simulated microgravity increased the expression of *ColIII*, *MMP1*, *CD44*, and *ITGB1*. ColIII is the main component in ECM of many extensible connective tissues such as skin, lung, uterus, intestine and the vascular system. It has a positive function in all stages of wound repair (32). Based on our results, it seems that impairment in the healing process in space is applied trough the effect of microgravity on different cellular or extracellular components. Moreover, ColIII is a molecular marker of fibroblast cells and it increases in fibroblastic differentiation. Thus, it may be suggested that simulated microgravity condition has a positive effect on the differentiation of MSCs toward fibroblast cells. We also used immunocytochemistry analysis to evaluate the expression of ColIII at protein level after 7 days exposure of ADSCs to simulated microgravity condition. Data of immunocytochemistry confirmed the results of real-time PCR, too.

Also, our data showed that ADSCs in simulated microgravity tended to form aggregated cell structures and grow in three- dimensional ways (Figure 5D). This is one of the features of simulated microgravity impact on cells that was also reported by others (17). The potential of three-dimensional growth of cells under simulated microgravity condition offers an opportunity for tissue engineering without using scaffold for regenerative medicine.

We also found that simulated microgravity increased the expression of *MMP1*. MMP1, also known as fibroblast collagenase which breaks down the interstitial collagens including ColIII. Surprisingly, we observed that simulated microgravity increased the expression of both *ColIII* and *MMP1*. There are some other studies that suggested *MMP1* and *ColIII *up-regulation together (33). Lee et al. (33) used connective tissue growth factor (CTGF) to differentiate MSCs into fibroblasts. They found that both *ColIII* and *MMP1* were up-regulated following CTGF induction. MMP1 is also a molecular marker of fibroblast cells and fibroblastic differentiation (33). Therefore, it may be emphasized again on the positive effect of simulated microgravity induction on the differentiation of MSCs into fibroblast cells. Therefore, it is likely to use simulated microgravity alone or in combination with a biochemical inducer for cell differentiation.

The expression of *CD44* and *ITGB1* was increased under simulated microgravity, too. CD44 is a cell surface adhesion molecule that participates in cellular signaling mechanisms through association with the actin polymer of the cytoskeleton, and can also interact with other molecules such as collagens and MMPs. It is an MSC marker that is involved in cell interactions, cell adhesion, cell migration, and homing (34). Our results are similar to that reported by Kumei et al. (35) during the real microgravity condition (space flight). One aspect of stem cell differentiation is down-regulation of stem cell markers during the differentiation process. However, upregulation of *CD44* antigen under simulated microgravity condition may be indicative of maintenance of an undifferentiated stage of ADSCs. This can lead to an increase of differential potential of ADSCs. Therefore, it can be speculated that pretreatment of ADSCs by simulated microgravity may increase the differentiation capacity of the cells. A similar conclusion was obtained by Yuge et al. (36).

We have also examined the mRNA expression of integrin subunit beta 1 (*ITGB1*). It is a membrane receptor involved in cell adhesion and signal transduction processes trough linking the cytoskeleton with the ECM. We observed that the expression of *ITGB1 *had increased after induction of simulated microgravity. Conflicting results reported in previous experiments may be due to differences in the microgravity environment, cell types and time of microgravity induction (16, 35, 37, 38). Similar to *MMP1* and *ColIII*, *ITGB1 *is also a molecular marker of fibroblast cells (39). Therefore, we suggest again the possible beneficial effect of simulated microgravity condition on the differentiation of ADSCs into fibroblast cells.

In contrast to above mentioned genes, simulated microgravity reduced the expression of *FBN1* and *VIM*. FBN1 is an extracellular matrix glycoprotein, and is essential for the formation of elastic fibers in the connective tissues to stabilize the ECM (40). VIM is the major cytoskeletal intermediate filament of mesenchymal cells that constitute the cytoskeleton along with tubulin microtubules and actin microfilaments, and maintains cell integrity (41). Previous studies have noted that simulated microgravity treatment reorganized the cytoskeleton, and resulted in a reduction of the main cytoskeletal elements. This would be one of the reasons for the changes in cell morphology and fate under simulated microgravity environment (19, 20). Our results were consistent with previous studies (20, 42).

In summary, we have examined the changes in the expression of some components of ECM and adhesion molecules in isolated human ADSCs under simulated microgravity condition. Our results revealed some alterations in the expression of selected genes after microgravity simulation. We believed that pretreatment of ADSCs by simulated microgravity may increase the differentiation capacity of these cells. Therefore, with a better understanding of this mechanical force and its effect on cells, it is likely to use simulated microgravity alone or in combination with the biochemical inducers for cell manipulation. Also, the potential of three-dimensional growth of cells under simulated microgravity condition offers an opportunity for tissue engineering without using scaffold for regenerative medicine.
